# Expression profiling of nasal polyp epithelial cells identifies two distinct phenotypes and suggests a role for neurogenic inflammation

**DOI:** 10.1186/2045-7022-5-S4-O5

**Published:** 2015-06-26

**Authors:** Marjolein Cornet, Kostamo Kristina, Rinia Bas, Zwinderman Koos, van Egmond Danielle, de Groot Esther, Fokkens Wytske, van Drunen Cornelis

**Affiliations:** 1 AMC, Otorhinolaryngology, Amsterdam, Netherlands; 2 AMC, Clinical Epidemiology, Amsterdam, Netherlands

## Background

Chronic rhinosinusitis with nasal polyps (CRSwNP) is a chronic inflammation of the nasal mucosa of unknown etiology. As airway epithelial cells have a well-accepted role in the regulation of innate defence and other local inflammatory processes we wanted to explore whether nasal polyp epithelial cells could play a role in the pathophysiology of CRSwNP.

## Method

Primary epithelial cells were isolated from nasal polyps of 24 affected individuals and from the middle turbinates of 9 healthy controls. After a limited culture period RNA was extracted and the expression profile determined using Human Genome U133 Plus 2.0 Genechip Array (Affymetrix inc., Santa Clara, CA, USA). Supernatants collected from the epithelial cells and immunohistochemistry on biopsies were used for validation.

## Results

The expression pattern in nasal polyp epithelial cells showed an aberrant expression for 23 genes compared to healthy controls. Furthermore, the expression pattern suggests two distinct epithelial profiles within the nasal polyposis group. Detailed analysis of these two distinct profiles reveals a deregulation of epithelial differentiation markers (KRTADP and CNFN) and of key regulators of neurogenic inflammation (SLURP1, LYNX1, and SLC44A4).

## Conclusion

Our data identified neurogenic inflammation as a potential novel player in the pathophysiology of CRSwNP and a possible dichrotomy of nasal polyp epithelial cells. The implications of these differences with healthy epithelial cells are not yet fully understood, but merit further investigation.

